# Efficacy of Therapeutic Aquatic Exercise vs Physical Therapy Modalities for Patients With Chronic Low Back Pain: A Randomized Clinical Trial

**DOI:** 10.1001/jamanetworkopen.2021.42069

**Published:** 2022-01-07

**Authors:** Meng-Si Peng, Rui Wang, Yi-Zu Wang, Chang-Cheng Chen, Juan Wang, Xiao-Chen Liu, Ge Song, Jia-Bao Guo, Pei-Jie Chen, Xue-Qiang Wang

**Affiliations:** 1 Department of Sport Rehabilitation, Shanghai University of Sport, Shanghai, China; 2 Department of Rehabilitation Medicine, The Second Affiliated Hospital of Hainan Medical University, Haikou, China; 3 Department of Rehabilitation Medicine, Qingtian People’s Hospital, Lishui, China; 4 Department of Rehabilitation Medicine, Changzhou Seventh People’s Hospital, Jiangsu Changzhou, China; 5 The Second School of Clinical Medicine, Xuzhou Medical University, Xuzhou, China; 6 Department of Rehabilitation Medicine, Shanghai Shangti Orthopaedic Hospital, Shanghai, China

## Abstract

**Importance:**

Therapeutic aquatic exercise is frequently offered to patients with chronic low back pain, but its long-term benefits are unclear.

**Objective:**

To assess the long-term effects of therapeutic aquatic exercise on people with chronic low back pain.

**Design, Setting, and Participants:**

This 3-month, single-blind randomized clinical trial with a 12-month follow-up period was performed from September 10, 2018, to March 12, 2019, and the trial follow-up was completed March 17, 2020. A total of 113 people with chronic low back pain were included in the experiment.

**Interventions:**

Participants were randomized to either the therapeutic aquatic exercise or the physical therapy modalities group. The therapeutic aquatic exercise group received aquatic exercise, whereas the physical therapy modalities group received transcutaneous electrical nerve stimulation and infrared ray thermal therapy. Both interventions were performed for 60 minutes twice a week for 3 months.

**Main Outcomes and Measures:**

The primary outcome was disability level, which was measured using the Roland-Morris Disability Questionnaire; scores range from 0 to 24, with higher scores indicating more severe disability. Secondary outcomes included pain intensity, quality of life, sleep quality, recommendation of intervention, and minimal clinically important difference. Intention-to-treat and per-protocol analyses were performed.

**Results:**

Of the 113 participants, 59 were women (52.2%) (mean [SD] age, 31.0 [11.5] years). Participants were randomly allocated into the therapeutic aquatic exercise group (n = 56) or the physical therapy modalities group (n = 57), and 98 patients (86.7%) completed the 12-month follow-up. Compared with the physical therapy modalities group, the therapeutic aquatic exercise group showed greater alleviation of disability, with adjusted mean group differences of −1.77 (95% CI, −3.02 to −0.51; *P* = .006) after the 3-month intervention, −2.42 (95% CI, −4.13 to −0.70; *P* = .006) at the 6-month follow-up, and −3.61 (95% CI, −5.63 to −1.58; *P* = .001) at the 12-month follow-up (*P* < .001 for overall group × time interaction). At the 12-month follow-up point, improvements were significantly greater in the therapeutic aquatic exercise group vs the physical therapy modalities group in the number of participants who met the minimal clinically important difference in pain (at least a 2-point improvement on the numeric rating scale) (most severe pain, 30 [53.57%] vs 12 [21.05%]; average pain, 14 [25%] vs 11 [19.30%]; and current pain, 22 [39.29%] vs 10 [17.54%]) and disability (at least a 5-point improvement on the Roland-Morris Disability Questionnaire) (26 [46.43%] vs 4 [7.02%]). One of the 56 participants (1.8%) in the therapeutic aquatic exercise group vs 2 of the 57 participants (3.5%) in the physical therapy modalities group experienced low back pain and other pains related to the intervention.

**Conclusions and Relevance:**

The therapeutic aquatic exercise program led to greater alleviation in patients with chronic low back pain than physical therapy modalities and had a long-term effect up to 12 months. This finding may prompt clinicians to recommend therapeutic aquatic exercise to patients with chronic low back pain as part of treatment to improve their health through active exercise rather than relying on passive relaxation.

**Trial Registration:**

Chinese Clinical Trial Registry: ChiCTR1800016396

## Key Points

**Question:**

Is therapeutic aquatic exercise as effective as physical therapy modalities in the management of adults with chronic low back pain?

**Findings:**

In this randomized clinical trial of 113 individuals with chronic low back pain, therapeutic aquatic exercise had a greater influence on pain, function, quality of life, sleep quality, and mental state than physical therapy modalities after a 3-month intervention, and the effect was present up to the 12-month follow-up. The recommendation rate of therapeutic aquatic exercise was significantly higher than that of physical therapy modalities.

**Meaning:**

The findings of this trial suggest that therapeutic aquatic exercise is an effective treatment for adults with chronic low back pain.

## Teaser

This randomized clinical trial evaluates the use of therapeutic aquatic exercise vs physical therapy in individuals with chronic low back pain.

## Introduction

Low back pain is a high-incidence and high-burden condition.^1^ The incidence rate of low back pain in a lifetime is 84% and that of chronic low back pain is approximately 23%.^2^ A systematic analysis of global burden of disease showed that the number of years lived by patients with low back pain disability increased by 17.5% between 2007 and 2017.^3^ In the US, the annual total direct expenses for each patient with chronic low back pain reached $8386.^4^ Clinical practice guidelines recommend therapeutic exercise and physical therapy modalities for low back pain.^5,6,7^ Therapeutic exercise and physical therapy modalities can relieve pain intensity and alleviate back disability for patients with low back pain.^8,9,10,11,12^ Transcutaneous electrical nerve stimulation and infrared ray thermal therapy are common modalities that are frequently used for treatment of chronic low back pain.^13,14^ Among the numerous therapeutic exercises available, therapeutic aquatic exercise is often prescribed by physicians for chronic low back pain, and it is becoming increasingly popular for treatment of chronic low back pain.^15,16^ Therapeutic aquatic exercise refers to water-based treatments or exercise. Water is an ideal environment for conducting an exercise program given its various properties, including buoyancy pressure, density, thermal capacity, and conductivity.^17,18,19,20,21,22^

Two systematic reviews suggested that therapeutic aquatic exercise can reduce pain intensity and improve function in patients with chronic low back pain.^23,24^ However, to our knowledge, evidence of the long-term benefits of therapeutic aquatic exercise in patients with chronic low back pain does not exist, and no study has compared the efficacy of therapeutic aquatic exercise and physical therapy modalities for chronic low back pain. Thus, we conducted a single-blind randomized clinical trial to compare the effects of therapeutic aquatic exercise with physical therapy modalities in patients with chronic low back pain over a 12-month follow-up period.

## Methods

### Study Design and Participants

We conducted a 3-month assessor-blinded randomized clinical trial with a 12-month follow-up period to compare the effects of therapeutic aquatic exercise and physical therapy modalities on chronic low back pain. All participants were included in a WeChat group for them to receive information about the trial. Regular offline health lectures were also held to offer educational information to the participants and carry out the measurements during the follow-up period. The protocol of this study was registered in the Chinese Clinical Trial Registry and is provided in Supplement 1. This study was approved by the ethics committee of the Shanghai University of Sport, Shanghai, China. Before enrolling in the project, all participants provided written informed consent. We recruited participants between September 10, 2018, and March 12, 2019. The trial follow-up was completed March 17, 2020. This study followed the Consolidated Standards of Reporting Trials (CONSORT) reporting guideline.

Inclusion criteria were age between 18 and 65 years; pain between the buttock band and the rib arch, with or without lower limb pain; pain intensity (when the most painful) of 3 or higher on a numeric rating scale; and chronic low back pain lasting at least 3 months. Exclusion criteria comprised mental illness or cognitive impairment, specific lumbago, regular low back pain exercise intervention during the past 6 months, pregnancy or lactation, chlorine allergy, and water-related anxiety or inability to adapt to an aquatic environment.

### Randomization and Blinding

A researcher who was separate from the intervention team selected 113 numbers from a certain position in the random number table and randomly divided them into the experimental and control groups. Then, the paper with numbers was placed into a sealed, opaque envelope. The envelopes were scrambled and numbered in turn. After inclusion, participants received the numbered envelopes consecutively and were divided into the corresponding groups according to the number on the paper within the envelope. Assessors were responsible for the measurements but unaware of the group assignments and remained distant from the intervention.

### Interventions

The intervention sessions were carried out by qualified physiotherapists (M.-S.P., Y.-Z.W., and C.-C.C.) who did not participate in data collection. Both programs lasted for 12 weeks and were administered for 60 minutes twice per week for a total of 24 treatment sessions. The participants were encouraged to complete the intervention as designed. The expected adherence rate was at least 75%.^25^ Attendance frequency and adverse events during the sessions were recorded on a daily record form. Once a participant was observed to be absent from an intervention session, they were contacted immediately to determine the reason for their absence. Participants who withdrew halfway, failed to attend the evaluations, or missed more than 2 weeks were considered to have dropped out.^25^

Participants in the therapeutic aquatic exercise group started the exercise with a 10-minute active warm-up session to enhance neuromuscular activation. Then, they performed an aquatic session for 40 minutes and had a 10-minute cool-down session. The target exercise intensity depended on the individual’s self-rated score of approximately 13, indicating 60% to 80% of their maximum heart rate on the Borg Scale Rating of Perceived Exertion, which is a measure sufficiently reliable for quantifying the training load for aquatic exercise.^26^ The participants in the physical therapy modalities group received transcutaneous electrical nerve stimulation and infrared ray thermal therapy. Both modalities were focused on pain points, and each had a duration of 30 minutes.^27,28,29,30^ Details of the interventions are presented in Supplement 1.

### Outcome Measures

Experienced physiotherapists carried out the measurements at baseline, after the 3-month intervention, at the 6-month follow-up, and at the 12-month follow-up from baseline. The primary outcome was the Roland-Morris Disability Questionnaire, which contains 24 items that are closely related to the daily life activities of patients with chronic low back pain.^31^ With scoring of 1 (yes) and 0 (no), the final score varies from 0 to 24. Higher scores are associated with more severe disability.^32^

The secondary outcome was a numeric rating scale, which consisted of 11 numbers from 0 to 10: 0 (painless), 1 to 3 (mild pain), 4 to 6 (moderate pain), and 7 to 10 (strong and unbearable pain). The participants reported the pain intensity they felt at the time of the report and that they experienced during the past week (slightest, average, and most serious).^33^

Considering that chronic low back pain may seriously affect the sleep quality of patients who have been experiencing pain for a long time and may lead to adverse psychological reactions, such as fear avoidance belief, we included the 36-item Short-form Health Survey,^34^ Self-rating Anxiety Scale,^35^ Zung Self-Rating Depression Scale,^36^ Pittsburgh Sleep Quality Index,^37^ Pain Anxiety Symptoms Scale,^38^ Tampa Scale for Kinesiophobia,^39,40^ Fear Avoidance Beliefs Questionnaire,^41^ minimal clinically important difference in pain and function,^42,43^ global perceived effect,^44,45^ adverse events, and participants’ recommendation levels on the intervention that they received as the secondary outcomes. Details on the outcome measures are presented in Supplement 1.

### Sample Size Calculation

Sample size was calculated by G*Power, version 3.1.9 (Heinrich-Heine-Universität Düsseldorf) based on the following conditions. As in the Costantino and Romiti^46^ trial, the participants in the intervention cohort received 3 months of therapeutic aquatic exercise, and those in the control group received the back school program. The effect size was calculated to be 0.35 by using the Roland-Morris Disability Questionnaire mean (SD) score of the intervention group (5.37 [1.82]) and the control group (6.11 [2.36]) during the 3-month follow-up. Results in the 2 groups were measured 4 times by using a mixed design of repeated-measures analysis of variance. Considering that α = .05, power (1-β) = 0.95, and correlation among repeated measures = 0.5, the total sample size was 70. Considering the possibility of a 20% missing rate, the minimum sample size was 88.

### Statistical Analysis

The data were collected and analyzed with Microsoft 2016 (Microsoft Corp) and SPSS, version 20.0 (IBM SPSS). In all analyses, statistical significance was accepted as *P* < .05 (2-tailed). For baseline variables, the χ^2^ test was used to test categorical variables (eg, sex and educational level), and the Mann-Whitney test was used to test continuous variables (eg, age and body mass index). The results are presented as number (percentage) or mean (SD).

The experimental results were compared through adjusted 2-way repeated-measures analysis of variance (group × time). The adjustment factors included sex, age, body mass index, physical activity, low back pain duration, numeric rating scale level of the most severe low back pain, medication, and smoking history. A χ^2^ test was conducted to compare the proportion of each group’s participants who met the minimal clinically important difference for pain and function at postintervention. Although the use of the minimal clinically important difference for the Roland-Morris Disability Questionnaire and numeric rating scale remains controversial, values of 5.0 and 2.0 are considered reasonable and are commonly used.^43^ The χ^2^ test was also applied to determine the difference between the 2 groups for the proportion of participants reporting global perceived effect, adverse events, and treatment recommendations.

Considering that some participants might drop out midway, all of the data were analyzed by using intention-to-treat analysis (including all randomized participants) and per-protocol analysis (participants who completed the intervention). Intention-to-treat was regarded as the primary analysis. Participants who withdrew from the intervention were contacted immediately to investigate their reasons for dropping out and were encouraged to continue the measurements to minimize the loss of follow-up data. If the participants failed to follow-up or withdrew from the group, their last observation results were carried forward to fill in the missing data for intention-to-treat analysis.

## Results

Of the 191 potential participants who were screened, 113 met all inclusion criteria and were randomly allocated into the therapeutic aquatic exercise group (n = 56) or the physical therapy modalities group (n = 57); of these, 98 patients (86.7%) completed the 12-month follow-up (Figure). The overall mean (SD) age of the participants was 31.0 (11.5) years, 54 were men (47.8%), and 59 were women (52.2%) (Table 1; eTable 1 in Supplement 2).

**Figure.**
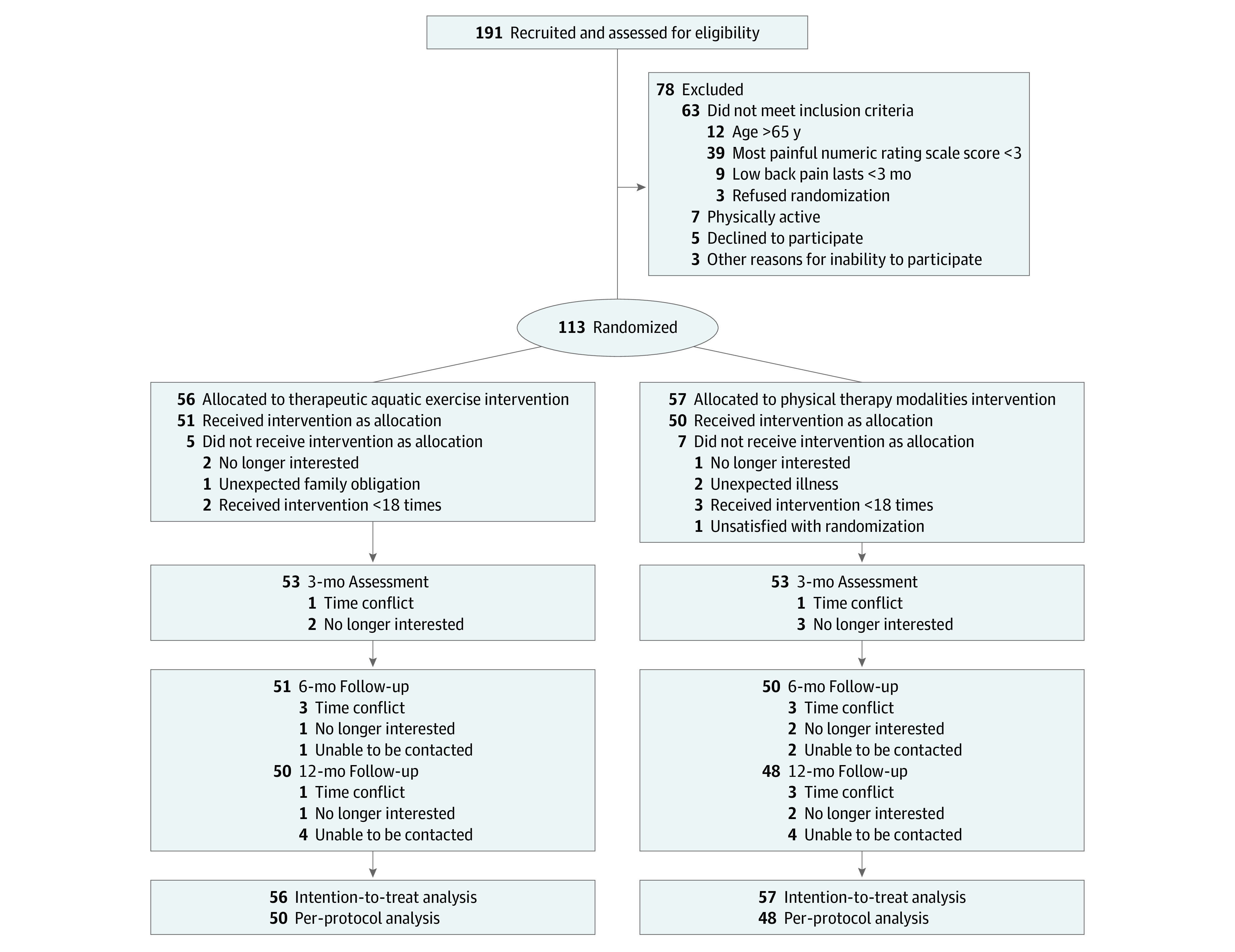
Flow Diagram of the Participants

**Table 1.** Baseline Demographic and Clinical Characteristics

Characteristic	Group, No. (%)	
	Therapeutic aquatic exercise (n = 56)	Physical therapy modalities (n = 57)
Age, mean (SD), y	31.7 (11.3)	30.4 (11.8)
Sex		
Male	30 (53.6)	24 (42.1)
Female	26 (46.4)	33 (57.9)
BMI, mean (SD)	23.19 (2.86)	22.94 (3.98)
Current back pain intensity, mean (SD)^a^		
Most serious pain in previous week	5.55 (1.28)	5.40 (1.49)
Average pain in previous week	3.96 (1.14)	4.02 (1.37)
Current pain intensity	2.70 (1.57)	2.72 (1.67)
Work absence or reduced hours, mean (SD), h	7.51 (23.90)	3.36 (12.06)
Medical expenditure on back pain last year, mean (SD), yuan^b^	0.54 (1.01)	0.37 (0.82)
Cause of first-onset pain		
Hyperactivity or improper exercise	23 (41.1)	21 (36.8)
Sedentary lifestyle	14 (25.0)	22 (38.6)
Pregnancy	0	2 (3.5)
Other	19 (33.9)	12 (21.1)
Site of first-onset low back pain		
Left	8 (14.3)	11 (19.3)
Right	12 (21.4)	14 (24.6)
Middle	21 (37.5)	13 (22.8)
Both sides	10 (17.9)	16 (28.1)
Other	5 (8.9)	3 (5.3)
Site of current low back pain		
Left	10 (17.9)	8 (14.0)
Right	13 (23.2)	11 (19.3)
Middle	18 (32.1)	14 (24.6)
Both sides	8 (14.3)	21 (36.8)
Other	7 (12.5)	3 (5.3)
Duration of the latest low back pain, mean (SD), d	12.66 (29.30)	13.82 (27.66)
Frequency of low back pain last month, mean (SD)	9.95 (8.71)	11.05 (9.90)
Duration of low back pain per day last week, mean (SD), h	7.04 (6.60)	5.82 (6.20)
Pain mode in 24 h		
Gradually aggravated	17 (30.4)	18 (31.6)
Gradually relieved	18 (32.1)	16 (28.1)
No change	13 (23.2)	11 (19.3)
Other	8 (14.3)	12 (21.1)
Factors aggravating low back pain		
Sitting	45 (80.4)	44 (77.2)
Standing	36 (64.3)	33 (57.9)
Walking	24 (42.9)	23 (40.4)
Bending	30 (53.6)	26 (45.6)
Squatting	10 (17.9)	9 (15.8)
Climbing stairs	3 (5.4)	7 (12.3)
Descending stairs	8 (14.3)	7 (12.3)
Postural change	5 (8.9)	2 (3.5)
Other	5 (8.9)	8 (14.0)
Factors to relieve low back pain		
Recumbent rest	46 (82.1)	43 (75.4)
Sitting rest	4 (7.1)	14 (24.6)
Small-intensity activities	22 (39.3)	17 (29.8)
Other	5 (8.9)	10 (17.5)
Nature of pain		
Soreness	41 (73.2)	44 (77.2)
Distended	19 (33.9)	23 (40.4)
Radiating	13 (23.2)	9 (15.8)
Burning	0	1 (1.8)
Needling	10 (17.9)	10 (17.5)
Other	2 (3.6)	0

Abbreviations: BMI, body mass index (calculated as weight in kilograms divided by height in meters squared); NRS, numeric rating scale.

^a^ Based on NRS score.

^b^ 1 US dollar = 6.3734 yuan.

### Outcomes

Compared with the participants in the physical therapy modalities group, those in the therapeutic aquatic exercise group showed improvement in disability by an additional −1.77 (95% CI, −3.02 to −0.51) points after the 3-month intervention, −2.42 (95% CI, −4.13 to −0.70) points at 6 months, and −3.61 (95% CI, −5.63 to −1.58) points at the 12-month follow-up (*P* < .001 for overall group × time interaction) (Table 2). Functional improvement was not significantly affected by age, sex, body mass index, low back pain duration, educational level, or pain level.

**Table 2.** Primary Outcomes at 3, 6, and 12 Months

RMDQ	Therapeutic aquatic exercise group (n = 56)	Physical therapy modalities group (n = 57)	Adjusted between-group difference, mean (95% CI)^a^	*P* value	*F* value	*P* value for overall group × time interaction
Baseline	8.82 (5.82)	8.37 (5.41)	NA	NA	8.28	<.001
3 mo	3.23 (2.90)	4.63 (3.98)	−1.77 (−3.02 to −0.51)	.006		
6 mo	3.55 (4.19)	5.61 (5.49)	−2.42 (−4.13 to −0.70)	.006		
12 mo	3.52 (4.43)	6.67 (6.47)	−3.61 (−5.63 to −1.58)	.001		

Abbreviations: NA, not applicable; RMDQ, Roland-Morris Disability Questionnaire.

^a^ Mean differences between groups were adjusted for sex, age, body mass index, physical activity, low back pain duration, numeric rating scale of the most severe low back pain, medication, and smoking history.

The secondary outcomes are presented in Table 3. Compared with the participants in the physical therapy modalities group, those in the therapeutic aquatic exercise group showed improvement in the most severe pain by an additional −0.79 (95% CI, −1.31 to −0.27) points after the 3-month intervention, −1.34 (95% CI, −2.06 to −0.62) points at 6 months, and −2.04 (95% CI, −2.75 to −1.34) points at the 12-month follow-up (*P* < .001 for overall group × time interaction). The slightest pain of the therapeutic aquatic exercise group improved by an additional −0.64 points after the 3-month intervention, −0.72 points at 6 months, and −1.17 at the 12-month follow-up (*P* = .005). All pain scores at each time point were significantly different between the 2 groups. The effect of the intervention on the most severe pain was significantly modified by the participants’ most severe pain level. Compared with the participants in the physical therapy modalities group, those in the therapeutic aquatic exercise group showed more improvements on the 36-item Short-form Health Survey (overall group × time interaction, *P* = .003), Pittsburgh Sleep Quality Index (overall group × time interaction, *P* = .02), Tampa Scale for Kinesiophobia (overall group × time interaction, *P* < .001), and Fear-Avoidance Beliefs Questionnaire (physical activity subscale overall group × time interaction, *P* = .04). These improvements were not influenced by age, sex, body mass index, low back pain duration, educational level, or pain level.

**Table 3.** Secondary Outcomes at 3, 6, and 12 Months

Outcome	Therapeutic aquatic exercise group (n = 56)	Physical therapy modalities group (n = 57)	Adjusted between-group difference, mean (95% CI)^a^	*P* value	*F* value	*P* value for overall group × time interaction
**NRS**						
Most severe						
Baseline	5.55 (1.28)	5.40 (1.49)	NA	NA	12.23	<.001
3 mo	2.70 (1.55)	3.39 (1.60)	−0.79 (−1.31 to −0.27)	.003		
6 mo	2.93 (1.52)	4.25 (2.19)	−1.34 (−2.06 to −0.62)	<.001		
12 mo	3.16 (1.66)	4.82 (2.21)	−2.04 (−2.75 to −1.34)	<.001		
Average						
Baseline	3.96 (1.14)	4.02 (1.37)	NA	NA	9.36	<.001
3 mo	1.64 (1.15)	2.47 (1.31)	−0.87 (−1.30 to −0.43)	<.001		
6 mo	2.07 (1.09)	3.30 (1.80)	−1.28 (−1.87 to −0.70)	<.001		
12 mo	2.27 (1.39)	3.72 (1.87)	−1.74 (−2.33 to −1.15)	<.001		
Current						
Baseline	2.70 (1.57)	2.72 (1.67)	NA	NA	7.31	<.001
3 mo	0.95 (1.00)	1.30 (1.32)	−0.52 (−0.94 to −0.09)	.02		
6 mo	1.55 (1.32)	1.89 (1.70)	−0.46 (−1.06 to −0.14)	.13		
12 mo	1.50 (1.32)	2.86 (1.89)	−1.65 (−2.28 to −1.02)	<.001		
**SF-36**						
Baseline	110.17 (12.76)	113.42 (12.01)	NA	NA	5.06	.003
3 mo	119.56 (14.17)	119.17 (12.45)	0.29 (−5.18 to 5.75)	.92		
6 mo	121.51 (13.04)	118.57 (12.92)	2.09 (−3.15 to 7.31)	.43		
12 mo	123.79 (12.27)	117.46 (15.84)	6.59 (0.82 to 12.35)	.03		
**SAS**						
Baseline	42.05 (8.64)	42.23 (9.79)	NA	NA	2.16	.09
3 mo	36.13 (7.16)	40.74 (10.81)	−4.09 (−7.80 to −0.39)	.03		
6 mo	40.64 (8.73)	41.46 (10.46)	−0.49 (−4.45 to 3.47)	.81		
12 mo	38.86 (7.50)	40.16 (10.46)	−2.10 (−5.78 to 1.58)	.26		
**SDS**						
Baseline	41.82 (9.07)	43.63 (9.84)	NA	NA	2.27	.09
3 mo	38.71 (9.54)	41.79 (11.46)	−3.79 (−8.08 to 0.51)	.08		
6 mo	39.16 (9.39)	45.81 (13.14)	−6.35 (−11.05 to −1.65)	.009		
12 mo	39.84 (8.72)	45.04 (13.75)	−5.32 (−10.04 to −0.60)	.03		
**PSQI**						
Baseline	7.04 (3.45)	6.91 (3.50)	NA	NA	3.45	.02
3 mo	5.21 (2.80)	6.11 (3.11)	−1.05 (−2.26 to 0.17)	.09		
6 mo	5.88 (3.23)	5.70 (3.69)	0.09 (−1.30 to 1.48)	.90		
12 mo	5.75 (2.59)	6.91 (3.42)	−1.32 (−2.56 to −0.09)	.04		
**PASS**						
Baseline	27.77 (12.25)	27.33 (12.77)	NA	NA	2.72	.06
3 mo	18.66 (10.36)	23.32 (12.94)	−4.86 (−9.29 to −0.43)	.03		
6 mo	17.36 (10.18)	22.07 (10.63)	−5.74 (−9.71 to −1.77)	.005		
12 mo	18.07 (13.91)	22.26 (14.25)	−4.33 (−9.80 to 1.15)	.12		
**TSK**						
Baseline	44.82 (5.70)	42.30 (4.99)	NA	NA	10.35	<.001
3 mo	38.91 (7.31)	40.81 (5.36)	−1.84 (−4.34 to 0.66)	.15		
6 mo	37.70 (9.18)	40.16 (5.61)	−2.81 (−5.76 to 0.13)	.06		
12 mo	37.84 (8.26)	41.12 (5.88)	−3.49 (−6.27 to −0.70)	.02		
**FABQ**						
FABQ-PA						
Baseline	12.29 (4.34)	12.58 (4.14)	NA	NA	2.86	.04
3 mo	9.05 (4.89)	11.25 (4.70)	−2.39 (−4.28 to −0.50)	.01		
6 mo	8.86 (4.72)	10.40 (5.09)	−1.94 (−3.85 to −0.02)	.048		
12 mo	7.71 (4.79)	10.82 (5.86)	−3.31 (−5.46 to −1.16)	.003		
FABQ-W						
Baseline	25.70 (9.46)	24.32 (9.16)	NA	NA	1.52	.21
3 mo	20.68 (11.10)	22.07 (11.06)	−1.38 (−5.70 to 2.94)	.53		
6 mo	20.96 (9.07)	22.61 (11.93)	−1.40 (−5.62 to 2.82)	.51		
12 mo	19.75 (10.38)	22.56 (11.24)	−1.88 (−6.10 to 2.33)	.38		
FABQ total						
Baseline	37.98 (11.83)	36.89 (11.48)	NA	NA	2.38	.07
3 mo	29.73 (14.94)	23.32 (13.86)	−3.77 (−9.34 to 1.81)	.18		
6 mo	29.82 (12.38)	33.02 (15.73)	−3.34 (−8.91 to 2.23)	.24		
12 mo	27.46 (14.22)	33.39 (15.17)	−5.19 (−10.95 to 0.56)	.08		

Abbreviations: FABQ, Fear-Avoidance Beliefs Questionnaire; FABQ-PA, FABQ physical activity; FABQ-W, FABQ work; NA, not applicable; NRS, numeric rating scale; PASS, Pain Anxiety Symptoms Scale; PSQI, Pittsburgh Sleep Quality Index; SAS, Self-Rating Anxiety Scale; SDS, Zung Self-rating Depression Scale; SF-36, 36-item Short-Form Health Survey; TSK, Tampa Scale for Kinesiophobia.

^a^ Mean differences between groups were adjusted for sex, age, body mass index, physical activity, low back pain duration, NRS of the most severe low back pain, medication, and smoking history.

For minimal clinically important difference, the number of participants who had at least a 2-point improvement on the numeric rating scale for the most severe pain differed between the 2 groups at all time points (3 months: odds ratio [OR], 5.24; *P* = .001; 6 months: OR, 3.68, *P* = .001; and 12 months: OR, 4.24; *P* < .001). For average pain, the percentage of patients who met the minimal clinically important difference varied between the 2 groups after 3 months of intervention (OR, 2.14; *P* = .048). For current pain, the percentage of patients who met the minimal clinically important difference varied between the 2 groups at the 12-month follow-up (OR, 3.04; *P* = .01). Moreover, the number of patients who met the minimal clinically important difference threshold for the disability significantly differed between the 2 groups at all time points (3 months: OR, 2.53; *P* = .02; 6 months: OR, 3.89; *P* = .001; and 12 months: OR, 11.48; *P* < .001) (eTable 2 in Supplement 2).

Two of 56 participants (3.6%) in the therapeutic aquatic exercise group vs 4 of the 57 participants (7.0%) in the physical therapy modalities group experienced low back pain and other pains related to the intervention. Several patients also experienced pain that was unrelated to the intervention. Characteristics of participants who reported adverse events were not different between the 2 groups. A total of 41 participants (73.2%) in the therapeutic aquatic exercise group and 37 participants (64.9%) in the physical therapy modalities group reported improvements in their low back pain symptoms after the 3-month intervention. Global perceived effect in the therapeutic aquatic exercise group was significantly better than that in the physical therapy modalities group (χ^2^ = 11.7; *P* = .03). Among the participants, 52 (92.9%) were willing to recommend therapeutic aquatic exercise to other patients with low back pain, whereas 44 (77.2%) were willing to recommend physical therapy modalities. The recommendation rates for the 2 treatments differed significantly (χ^2^ = 9.5, *P* = .01). (Table 4).

**Table 4.** Adverse Events, GPE, and Recommendation of Participants After 3 Months of Intervention

Measure	Group, No. (%)		χ^2^ value	*P* value
	Therapeutic aquatic exercise (n = 56)	Physical therapy modalities (n = 57)		
Adverse events				
Low back pain related to intervention				
Yes	1 (1.8)	2 (3.5)	1.38	.61
No	52 (92.9)	54 (94.7)		
Don't know	3 (5.4)	1 (1.8)		
Low back pain unrelated to intervention				
Yes	7 (12.5)	8 (14.0)	0.16	.92
No	44 (78.6)	43 (75.4)		
Don't know	5 (8.9)	6 (10.5)		
Other pain related to intervention				
Yes	1 (1.8)	2 (3.5)	0.68	.77
No	53 (94.6)	52 (91.2)		
Don't know	2 (3.6)	3 (5.3)		
Other pain unrelated to intervention				
Yes	6 (10.7)	4 (7.0)	1.38	.63
No	50 (89.3)	52 (91.2)		
Don't know	0	1 (1.8)		
Global perceived effect				
Very much improved	5 (8.9)	0	11.67	.03
Much improved	14 (25.0)	7 (12.3)		
Minimally improved	22 (39.3)	30 (52.6)		
No change	14 (25.0)	18 (31.6)		
Minimally worse	0	1 (1.8)		
Much worse	1 (1.8)	0		
Very much worse	0	1 (1.8)		
Recommendation				
Highly recommended	15 (26.8)	6 (10.5)	9.46	.01
Recommended	37 (66.1)	38 (66.7)		
Unclear	3 (5.4)	12 (21.1)		
Not recommended	1 (1.8)	1 (1.8)		
Strongly deprecated	0	0		

Abbreviation: GPE, global perceived effect.

The results of per-protocol analysis are included in eTable 3 and eTable 4 in Supplement 2. An intention-to-treat analysis using the worst case of the participants’ group was performed to minimize the bias of follow-up; the results are presented in eTable 5 in Supplement 2.

## Discussion

The intention-to-treat analyses of the3-month intervention trial and 12-month follow-up of therapeutic aquatic exercise vs physical therapy modalities for chronic low back pain showed that the participants in the therapeutic aquatic exercise group gained significantly greater and more clinically meaningful improvement in disability compared with improvement in the physical therapy modalities group. We also found that therapeutic aquatic exercise was a more effective treatment than physical therapy modalities on pain intensity, quality of life, sleep quality, kinesiophobia, and fear avoidance for patients with chronic low back pain.

Shi et al^23^ published a meta-analysis that included 8 randomized clinical trials of therapeutic aquatic exercise for chronic low back pain. The duration of the therapeutic aquatic exercise intervention was 4 to 15 weeks. The intervention was administered 2 to 5 times a week, each for 30 to 80 minutes. The results suggested that therapeutic aquatic exercise could significantly reduce the pain intensity of patients with chronic low back pain and improve their functional level. Consistent with these results, our findings showed that the improvement in pain and dysfunction in the therapeutic aquatic exercise group was not only statistically significant but was also clinically significant. Baena-Beato et al^47^ and Bronwyn^48^ divided patients with chronic low back pain into the therapeutic aquatic exercise group and the waiting group and found that therapeutic aquatic exercise could significantly improve pain degree, dysfunction level, and quality of life. In addition, therapeutic aquatic exercise had a good effect on the anxiety level of patients with chronic low back pain. Sugano and Nomura^49^ reported that therapeutic aquatic exercise lowered the anxiety level in patients with chronic low back pain and, in a study by Bayraktar et al,^50^ most patients had high adherence to therapeutic aquatic exercise and were willing to recommend this treatment to others.

To our knowledge, the efficacy of therapeutic aquatic exercise in the treatment of patients with chronic low back pain has never been compared with that used in our control group of physical therapy modalities. Some researchers who chose to carry out similar exercises in water and on land discovered that the efficiency of therapeutic aquatic exercise and degree of pain relief associated with therapeutic aquatic exercise were better than those of land exercise.^19,51,52^ Even after a single intervention, the frequency of pain in the therapeutic aquatic exercise group was reduced to half of that in the land exercise group. Participants in the therapeutic aquatic exercise group also experienced greater improvement in quality of life and dysfunction than those in the land exercise group. Other researchers provided treatment, such as conventional physical therapy (low back pain pamphlet and lumbar exercise), multimodal physical therapy, or low back pain school, as an intervention in the control group.^46,53,54^ The dysfunction and quality of life of patients with chronic low back pain significantly improved after the therapeutic aquatic exercise intervention was added to the treatments.

### Strengths and Limitations

Our study had several strengths. To our knowledge, this was the first study to compare the efficacy of therapeutic aquatic exercise and physical therapy modalities in the treatment of chronic low back pain. The control treatment consisted of transcutaneous electrical nerve stimulation and infrared ray thermal therapy, which are widely used in clinical practice and are proven to exert good curative effects in patients with chronic low back pain. Second, self-reported information related to chronic low back pain was recorded in detail, and basic information, such as age, sex, low back pain duration, and pain level, which might affect the results, were selected as covariates in statistical analysis. Third, considering that differences between adherent and nonadherent participants might influence the results, intention-to-treat analysis and per-protocol analysis were performed. Both analyses supported the fact that therapeutic aquatic exercise was better than physical therapy modalities in some measured aspects. Fourth, this work had a long follow-up period and a large experimental sample, which ensured adequate statistical power for detecting the minimal clinical and long-term effects.

This study also had several limitations. First, although the age range was limited to 18 to 65 years, most of the participants were younger. Therefore, a stratified age design should be considered for future studies. Second, the self-reported pain level was low. Thus, the research results may not be generalizable to the whole population of people with chronic low back pain. The benefits and safety of therapeutic aquatic exercise for people with high levels of low back pain warrant study. Thus, individuals with different pain levels should be considered in future research. Third, given that the therapeutic aquatic exercise group received aquatic exercise and the physical therapy modalities group did not receive exercise, whether the effect of therapeutic aquatic exercise originated from the benefits of the water environment or from the benefits of active exercise was unclear. Future studies designed to include a group that receives land exercise to reflect the benefits of therapeutic aquatic exercise on chronic low back pain are needed. Fourth, combining therapeutic aquatic exercise and physical therapy modalities might be a better rehabilitation program for patients with chronic low back pain—this is widely used in some rehabilitation centers. Further studies could design a 3-group comparison. Fifth, blinding of participants and interventionists was impossible. Sixth, whether the medical costs and productivity losses of the 2 treatment options differed was unclear; socioeconomic indicators can be included for analysis in future works.

## Conclusions

In this randomized clinical trial, patients with chronic low back pain who received therapeutic aquatic exercise showed greater improvement in terms of function, pain, quality of life, sleep quality, and mental state than those who underwent physical therapy modalities. Therapeutic aquatic exercise is a safe treatment for chronic low back pain and most participants who received it were willing to recommend it to other patients with chronic lowe back pain.

## Notes

Supplement 1.
Trial Protocol

Supplement 2. **eTable 1.** Demographic and Clinical Characteristics of Participants in Each Group at Baseline **eTable 2.** The Number and Percentage of Participants Who Met MCID for Pain Level and Functional Status at 3, 6 and 12 Months **eTable 3.** Primary Outcomes for the Two Groups at 3, 6, and 12 Months (Per-Protocol Analysis) **eTable 4.** Secondary Outcomes for the Two Groups at 3, 6, and 12 Months (Per-Protocol Analysis) **eTable 5.** Outcomes for the Two Groups at 3, 6, and 12 Months (Intention-to-Treat Analysis With the Worst Case)

Supplement 3.
Data Sharing Statement
