# Errors in Table 3, Outcome Measures, Results, Supplement 1, and Supplement 2

**DOI:** 10.1001/jamanetworkopen.2024.9399

**Published:** 2024-03-29

In the Original Investigation titled, “Efficacy of Therapeutic Aquatic Exercise vs Physical Therapy Modalities for Patients With Chronic Low Back Pain: A Randomized Clinical Trial,”^1^ published January 7, 2022, there were errors in the article text, Table 3, and Supplements 1 and 2. First, a numeric error appeared in Table 3. The value for FABQ total category at 3 months in the physical therapy modalities group given as “23.32” should have been “33.32.” In addition, the trial protocol in Supplement 1 included pain as a primary outcome, which was not consistent with the published article. Other secondary outcomes were missing from the Outcomes Measures. The minimal clinically important findings in Results required updates. Finally, eTable 1 of Supplement 2 categories were given incorrectly with results of statistical tests that should not have been included for baseline characteristics, and eTable 2 contained entry errors on the rating scale for assessment of pain. The authors have explained what happened in a Comment,^2^ and this article has been corrected with updated Supplement materials.^1^
