# Supplementary material for: Efficacy of Therapeutic Aquatic Exercise vs Physical Therapy Modalities for Patients With Chronic Low Back Pain: A Randomized Clinical Trial
Source: JAMA Netw Open. 2022 Jan 7;5(1):e2142069. doi: 10.1001/jamanetworkopen.2021.42069 (PMC8742191; doi:10.1001/jamanetworkopen.2021.42069)
Supplement: Supplement 2. — eTable 1. Demographic and Clinical Characteristics of Participants in Each Group at Baseline eTable 2. The Number and Percentage of Participants Who Met MCID for Pain Level and Functional Status at 3, 6 and 12 Months eTable 3. Primary Outcomes for the Two Groups at 3, 6, and 12 Months (Per-Protocol Analysis) eTable 4. Secondary Outcomes for the Two Groups at 3, 6, and 12 Months (Per-Protocol Analysis) eTable 5. Outcomes for the Two Groups at 3, 6, and 12 Months (Intention-to-Treat Analysis With the Worst Case) [file jamanetwopen-e2142069-s002.pdf]

Supplemental Online Content

Peng MS, Wang R, Wang YZ, et al. Efficacy of therapeutic aquatic exercise vs physical therapy modalities for patients with chronic low back pain: a randomized clinical trial. *JAMA Netw Open*. 20215(1):e2142069. doi:10.1001/jamanetworkopen.2021.42069

eTable 1. Demographic and Clinical Characteristics of Participants in Each Group at Baseline

eTable 2. The Number and Percentage of Participants Who Met MCID for Pain Level and Functional Status at 3, 6 and 12 Months

eTable 3. Primary Outcomes for the Two Groups at 3, 6, and 12 Months (Per-Protocol Analysis)

eTable 4. Secondary Outcomes for the Two Groups at 3, 6, and 12 Months (Per-Protocol Analysis)

eTable 5. Outcomes for the Two Groups at 3, 6, and 12 Months (Intention-to-Treat Analysis With the Worst Case)

This supplemental material has been provided by the authors to give readers additional information about their work.

© 2021 Peng MS et al. *JAMA Network Open.*

eTable1 Demographic and clinical characteristics of participants in each group at baseline

Characteristic	Therapeutic aquatic exercise group (n=56)	physical therapy modalities group (n=57)	Z/χ2	P Value
Age, mean (SD), y	31.72（11.32）	30.36（11.76）	-1.169	0.243*
Sex-male	30（53.6）	24（42.1）	1.488	0.222#
Height, mean (SD), m	168.45（7.92）	167.53（8.71）	-0.517	0.605*
Weight, mean (SD), kg	66.22（11.91）	64.85（14.43）	-0.836	0.403*
BMI, mean(SD), kg/m^2	23.19（2.86）	22.94（3.98）	-1.321	0.187*
**Education levels**				
Illiteracy, No. (%)	0（0）	0（0）		
Primary school, No. (%)	0（0）	1（1.75）		
Junior middle school, No. (%)	0（0）	1（1.75）		
High school, No. (%)	3（5.36）	1（1.75）	3.949	0.353#
University, No. (%)	31（55.36）	26（45.61）		
Postgraduate, No. (%)	22（39.29）	28（49.12）		
**Employment status**				
Employed part-time, No. (%)	2（3.57）	5（8.77）		
Employed full-time, No. (%)	24（42.86）	16（28.07）		
Unemployed, No. (%)	0（0）	0（0）		
Not trying to look for employment, No. (%)	0（0）	0（0）	3.988	0.263#
Unable to work due to poor health, No. (%)	0（0）	0（0）		
Student, No. (%)	28（50）	35（61.4）		
Retired, No. (%)	2（3.57）	1（1.75）		
**Personal monthly income, yuan**				
⩾10000, No. (%)	20（35.71）	21（36.84）		
5000-10000, No. (%)	7（12.50）	14（24.56）		0.174#
3000-5000, No. (%)	5（8.93）	6（10.53）	6.350	
＜3000, No. (%)	15（26.79）	6（10.53）		

© 2021 Peng MS et al. *JAMA Network Open.*

Refused, No. (%)	9（16.07）	10（17.54）		
**Smoking history**				
Smoking, No. (%)	10（17.9）	10（17.5）	0.002	0.965#
Years of smoking, mean (SD), y	1.68（4.77）	0.98（4.10）	-0.341	0.733*
Smoking per day, mean (SD)	1.59（4.85）	0.91（2.28）	-0.095	0.924*
Low back pain duration, mean (SD), y	6.21（5.65）	7.28（7.34）	-0.083	0.934*
Duration of first onset, mean (SD),d	24.02（32.55）	22.09（33.58）	-0.968	0.333*
**Back pain intensity**				
Most serious pain in previous week, mean NRS score (SD)	5.55(1.28)	5.40(1.49)	0.575	0.566*
Slightest pain in previous week, mean NRS score (SD)	2.27(1.17)	2.19(1.17)	0.340	0.734*
Work absence or reduced hours, mean (SD), h	7.51（23.90）	3.36（12.06）	-1.823	0.068*
Medical expenditure on back pain last year, mean (SD), yuan	0.54（1.01）	0.37（0.82）	-0.672	0.501*
**Medication use in previous three months**				
No medication, No. (%)	42（75）	40（70.18）	3.938	0.269#
Pain reliever, No. (%)	7（12.50）	6（10.53）		
Adjuvant drugs, No. (%)	8（8.93）	13（22.81）		
Drugs for other disease, No. (%)	2（3.57）	1（1.75）		
**Belief that invention works**				
Yes, No. (%)	34（60.71）	40（70.18）	1.119	0.290#
No, No. (%)	0（0）	0（0）		
Don't know, No. (%)	22（39.29）	17（29.82）		
**Expectation that invention works**				
Yes, No. (%)	55（98.21）	56（98.25）	0.000	1.000#
No, No. (%)	0（0）	0（0）		
Don't know, No. (%)	1（1.79）	1（1.75）		
**Marital status**				
Unmarried, No. (%)	33（58.93）	38（66.67）	0.986	0.714#
Married, No. (%)	22（39.29）	18（31.58）		

© 2021 Peng MS et al. *JAMA Network Open.*

Divorced, No. (%)	1（1.79）	1（1.75）		
Widowhood, No. (%)	0（0）	0（0）		
**Occupation**	8.45（2.76）	8.93（2.80）	-1.204	0.229[*]
Sitting time , mean (SD), h	5.88（2.78）	6.70（3.15）	-1.234	0.217[*]
Standing time, mean (SD), h	2.05（2.06）	1.89（1.59）	-1.000	0.920[*]
Others, mean (SD), h	0.54（1.01）	0.37（0.82）	-0.819	0.413[*]
**Physical activity**				
**High intensity physical activity per week**				
＜150min per day, No. (%)	24（42.86）	28（49.12）		
150-300min per day, No. (%)	16（28.57）	12（21.05）	0.073	0.964[#]
＞300min per day, No. (%)	16（28.57）	17（29.82）		
**Moderate intensity physical activity per week**				
＜75min per day, No. (%)	19（33.93）	18（31.58）		
75-150min per day, No. (%)	15（26.79）	16（28.07）	0.901	0.637[#]
＞150min per day, No. (%)	22（39.29）	23（40.35）		
**Cause of first onset**				
Hyperactivity or improper exercise, No. (%)	23（41.07）	21（36.84）		
Sedentary lifestyle, No. (%)	14（25.00）	22（38.60）	5.031	0.140[#]
Pregnancy, No. (%)	0（0）	2（3.51）		
Others, No. (%)	19（33.93）	12（21.05）		
**Site of first onset**				
Left, No. (%)	8（14.29）	11（19.30）		
Right, No. (%)	12（21.43）	14（24.56）		
Middle, No. (%)	21（37.5）	13（22.81）	4.363	0.368[#]
Both two sides, No. (%)	10（17.86）	16（28.07）		
Others, No. (%)	5（8.93）	3（5.26）		
**Site of current LBP**				
Left, No. (%)	10（17.86）	8（14.04）	8.281	0.080[#]
Right, No. (%)	13（23.21）	11（19.30）		

© 2021 Peng MS et al. *JAMA Network Open.*

Middle, No. (%)	18（32.14）	14（24.56）		
Both two sides, No. (%)	8（14.29）	21（36.84）		
Others, No. (%)	7（12.50）	3（5.26）		
Duration of the latest low back pain, mean (SD),d	12.66（29.26）	13.82（27.66）	-1.095	0.273*
Frequency of low back pain last month, mean (SD)	9.95（8.71）	11.05（9.90）	-0.231	0.817*
Duration of low back pain per day last week, mean (SD),h	7.04（6.60）	5.82（6.20）	-1.354	0.176*
**Influence of low back pain on work**				
Free, No. (%)	5（8.93）	6（10.53）		
mild, No. (%)	32（57.14）	36（63.16）	2.057	0.595#
moderate, No. (%)	15（26.79）	14（24.56）		
severe, No. (%)	4（7.14）	1（1.75）		
**Influence of low back pain on life**				
Free, No. (%)	2（3.57）	5（8.77）		
mild, No. (%)	34（60.71）	40（70.18）	4.259	0.227#
moderate, No. (%)	16（28.57）	11（19.30）		
severe, No. (%)	4（7.14）	1（1.75）		
**Pain mode in 24-hour**				
gradually aggravate, No. (%)	17（30.36）	18（31.58）		
gradually relieve, No. (%)	18（32.14）	16（28.07）	1.104	0.776#
no change, No. (%)	13（23.21）	11（19.30）		
Others, No. (%)	8（14.29）	12（21.05）		
**Factors aggravating low back pain**				
Sitting, No. (%)	45（80.36）	44（77.19）		
Standing, No. (%)	36（64.29）	33（57.89）		
Walking, No. (%)	24（42.86）	23（40.35）		
Bending, No. (%)	30（53.57）	26（45.61）	3.934	0.875#
Squat down, No. (%)	10（17.86）	9（15.79）		
Go upstairs, No. (%)	3（5.36）	7（12.28）		
Go downstairs, No. (%)	8（14.29）	7（12.28）		

© 2021 Peng MS et al. *JAMA Network Open.*

Postural change, No. (%)	5（8.93）	2（3.51）		
Others, No. (%)	5（8.93）	8（14.04）		
**Factors to relieve low back pain**				
Recumbent rest, No. (%)	46（82.14）	43（75.44）		
Sitting for rest, No. (%)	4（7.14）	14（24.56）	7.675	0.053#
Small intensity activities, No. (%)	22（39.29）	17（29.82）		
Others, No. (%)	5（8.93）	10（17.54）		
**Nature of pain**				
Soreness, No. (%)	41（73.21）	44（77.19）		
Distended pain, No. (%)	19（33.93）	23（40.35）		
Radiation pain, No. (%)	13（23.21）	9（15.79）	3.779	0.607#
Burning pain, No. (%)	0（0）	1（1.75）		
Needling pain, No. (%)	10（17.86）	10（17.54）		
Other, No. (%)	2（3.57）	0（0）		

Note: *Mann-Whitney U test, #Chi-square test.

© 2021 Peng MS et al. *JAMA Network Open.*

eTable2 The number and percentage of participants who met MCID for pain level and functional status at 3, 6 and 12 months.

Measure	Therapeutic aquatic exercise group (n=56)	physical therapy modalities group (n=57)	Odd ratio (95%CI)	$\chi 2$	P Value
NRS of the most severe pain					
3 mo	43(76.79)	27(47.37)	5.24(1.93 to 14.25)	11.782	0.001
6 mo	31(55.36)	20(35.09)	3.68(1.64 to 8.26)	10.370	0.001
12 mo	30(53.57)	12(21.05)	4.24(1.93 to 9.33)	13.518	< 0.001
NRS of average pain					
3 mo	30(53.57)	20(35.09)	2.14(1.00 to 4.55)	3.912	0.048
6 mo	16(28.57)	10(17.54)	1.88(0.77 to 4.60)	1.939	0.164
12 mo	14(25.00)	11(19.30)	1.39(0.57 to 3.41)	0.533	0.465
NRS of current pain					
3 mo	33(58.93)	25(43.86)	1.84(0.87 to 3.87)	2.568	0.109
6 mo	22(39.29)	24(42.11)	0.89(0.42 to 1.89)	0.093	0.760
12 mo	22(39.29)	10(17.54)	3.04(1.28 to 7.25)	6.578	0.010
RMDQ					
3 mo	29(51.79)	17(29.82)	2.53(1.17 to 5.47)	5.644	0.018
6 mo	27(48.21)	11(19.30)	3.89(1.68 to 9.03)	10.582	0.001
12 mo	26(46.43)	4(7.02)	11.48(3.66 to 36.05)	22.500	< 0.001

© 2021 Peng MS et al. *JAMA Network Open.*

eTable3 Primary outcomes for the two Groups at 3, 6, and 12 Months (per-protocol analysis).

RMDQ	Therapeutic aquatic exercise group (n=50)	physical therapy modalities group (n=48)	Adjusted Between-Group Difference, Mean (95%CI) [a]	P Value	F Value	p Value for Overall Group * Time Interaction
Baseline	8.84(6.04)	8.31(5.47)				
3 mo	3.24(3.00)	4.23(4.05)	−1.30(−2.67 to 0.07)	0.063	7.529	＜0.001
6 mo	3.58(4.17)	5.56(5.78)	−2.26(−4.15 to −0.38)	0.019		
12 mo	3.52(4.39)	6.52(6.44)	−3.42(−5.56 to −1.28)	0.002		

Abbreviation: Abbreviation: Roland Morris disability questionnaire (RMDQ).

[a] Mean difference between groups were adjusted for gender, age, BMI, physical activity, LBP duration, NRS of the most severe LBP, medication, and smoking history.

© 2021 Peng MS et al. *JAMA Network Open.*

eTable4 Secondary outcomes for the two Groups at 3, 6, and 12 Months (per-protocol analysis).

	Therapeutic aquatic exercise group (n=50)	physical therapy modalities group (n=48)	Between-Group Difference(95%CI)	P Value	F Value	p Value for Overall Group * Time Interaction
**NRS**						
Baseline	5.56(1.34)	5.35(1.47)				
3 mo	2.68(1.52)	3.29(1.47)	−0.79(−1.31 to −0.27)	0.003	12.171	<0.001
6 mo	2.86(1.47)	4.25(2.12)	−1.37(−2.13 to −0.62)	<0.001		
12 mo	3.14(1.65)	4.88(2.10)	−2.13(−2.87 to −1.39)	<0.001		
Average						
Baseline	3.86(1.14)	4.00(1.32)				
3 mo	1.64(1.14)	2.42(1.27)	−0.87(−1.32 to −0.42)	<0.001	7.467	<0.001
6 mo	2.06(1.08)	3.29(1.73)	−1.29(−1.90 to −0.68)	<0.001		
12 mo	2.26(1.38)	3.71(1.79)	−1.78(−2.40 to −1.15)	<0.001		
Current						
Baseline	2.62(1.58)	2.63(1.67)				
3 mo	0.94(1.02)	1.13(1.20)	−0.43(−0.86 to −0.01)	0.048	5.573	0.002
6 mo	1.62(1.35)	1.90(1.64)	−0.41(−1.05 to 0.22)	0.198		
**SF-36**						
Baseline	110.56(12.14)	113.60(12.20)				
3 mo	118.29(14.39)	119.53(13.18)	-1.49(-7.58 to 4.59)	0.627	2.524	0.063
6 mo	120.74(13.60)	120.11(12.94)	-0.26(-6.02 to 5.50)	0.929		
12 mo	122.89(12.64)	118.19(16.47)	5.28(-1.12 to 11.67)	0.105		
**SAS**						
Baseline	41.45(7.70)	42.37(10.06)				
3 mo	35.45(6.61)	41.19(11.00)	-5.60(-9.56 to -1.64)	0.006	4.877	0.004
6 mo	40.31(8.34)	40.52(9.79)	-0.21(-4.23 to 3.81)	0.917		
12 mo	38.89(7.54)	39.77(10.84)	-1.72(-5.79 to 2.35)	0.403		
**SDS**						
Baseline	41.77(8.95)	43.81(10.02)				
3 mo	38.65(9.70)	42.09(11.46)	-4.30(-8.95 to 0.35)	0.070	1.305	0.278
6 mo	38.99(9.35)	45.00(12.96)	-5.84(-10.84 to -0.85)	0.022		

© 2021 Peng MS et al. *JAMA Network Open.*

12 mo	39.25(8.52)	44.69(14.15)	-5.36(-10.52 to -0.21)	0.042		
**PSQI**						
Baseline	6.95(3.38)	7.03(3.60)				
3 mo	5.36(2.85)	6.02(3.21)	-0.88(-2.21 to 0.46)	0.196	3.889	0.012
6 mo	5.98(2.99)	5.54(3.80)	0.19(-1.28 to 1.67)	0.796		
12 mo	5.66(2.30)	6.73(3.55)	-1.35(-2.64 to -0.05)	0.042		
**PASS**						
Baseline	26.76(12.17)	27.02(11.66)				
3 mo	18.94(10.40)	23.17(13.44)	-4.05(-8.88 to 0.79)	0.100	1.283	0.285
6 mo	18.02(10.37)	21.67(10.69)	-4.64(-8.97 to -0.32)	0.036		
12 mo	17.88(11.70)	22.44(15.18)	-4.88(-10.39 to 0.63)	0.082		
**TSK**						
Baseline	44.68(5.94)	42.19(4.45)				
3 mo	38.98(7.68)	40.60(5.34)	-1.33(-4.11 to 1.44)	0.342	8.203	＜0.001
6 mo	37.96(9.39)	40.13(5.52)	-2.59(-5.82 to 0.65)	0.116		
12 mo	37.90(8.71)	40.96(6.05)	-3.27(-6.41 to -0.13)	0.041		
**FABQ**						
**FABQ-PA**						
Baseline	11.94(4.10)	12.33(3.97)				
3 mo	9.22(4.85)	10.88(4.77)	-1.83(-3.86 to 0.19)	0.076	1.698	0.168
6 mo	8.64(4.82)	9.94(5.05)	-1.64(-3.65 to 0.37)	0.108		
12 mo	7.64(4.53)	10.33(6.17)	-2.93(-5.26 to -0.61)	0.014		
**FABQ-W**						
Baseline	25.10(9.51)	24.67(9.18)				
3 mo	20.92(11.30)	21.69(11.58)	-0.90(-5.76 to 3.96)	0.713	0.325	0.807
6 mo	20.54(9.25)	21.73(12.34)	-1.09(-5.77 to 3.59)	0.644		
12 mo	19.88(10.19)	21.85(11.50)	-1.17(-5.76 to 3.43)	0.615		
**FABQ-TOTAL**						
Baseline	37.04(11.55)	37.00(11.26)				
3 mo	30.14(15.02)	32.56(14.37)	-2.73(-8.92 to 3.45)	0.382	0.860	0.462
6 mo	29.18(12.54)	31.67(16.13)	-2.73(-8.80 to 3.34)	0.374		
12 mo	27.52(13.79)	32.19(15.62)	-4.10(-10.37 to 2.18)	0.197		

© 2021 Peng MS et al. *JAMA Network Open.*

Abbreviation: Numeric Rating Scale(NRS), Short Form Health Survey(SF-36), Self-Rating Anxiety Scale (SAS), Zung Self-Rating Depression Scale (SDS), Pittsburgh Sleep Quality Index (PSQI), Pain Anxiety Symptoms Scale (PASS), Tampa Scale for Kinesiophobia (TSK), Fear Avoidance Beliefs Questionnaire（FABQ）

[a] Mean difference between groups were adjusted for gender, age, BMI, physical activity, LBP duration, NRS of the most severe LBP, medication, and smoking history

© 2021 Peng MS et al. *JAMA Network Open.*

eTable5 Outcomes for the two Groups at 3, 6, and 12 Months(Intention- to treat analysis with the worst case)

	Therapeutic aquatic exercise group (n=50)	physical therapy modalities group (n=48)	Between-Group Difference(95%CI)	P Value	F Value	p Value for Overall Group * Time Interaction
**RMDQ**						
Baseline	8.82(5.82)	8.37(5.41)		0.959	4.893	0.002
3 mo	3.89(3.75)	5.26(4.69)	-1.57(-3.15 to -0.01)	0.051		
6 mo	4.57(5.24)	7.40(7.65)	-3.66(-6.17to -1.15)	0.005		
12 mo	4.38(5.49)	7.00(6.86)	-3.29(-5.67 to -0.92)	0.007		
**NRS**						
Most severe						
Baseline	5.55(1.28)	5.40(1.49)			10.573	＜0.001
3 mo	2.90(1.83)	3.65(1.97)	-0.79(-1.44 to -1.26)	0.020		
6 mo	3.13(1.71)	4.91(2.72)	-1.93(-2.83 to -1.04)	0.000		
12 mo	3.41(1.91)	4.96(2.22)	-2.00(-2.76 to -1.25)	0.000		
Average						
Baseline	3.96(1.14)	4.02(1.37)			8.335	＜0.001
3 mo	1.86(1.51)	2.77(1.74)	-0.88(-1.47 to -0.28)	0.004		
6 mo	2.18(1.16)	3.82(2.22)	-1.77(-2.48 to -1.07)	0.000		
12 mo	2.46(1.55)	3.84(1.92)	-1.71(-2.35 to -1.07)	0.000		
Current						
Baseline	2.70(1.57)	2.72(1.67)			4.584	0.005
3 mo	1.09(1.20)	1.42(1.53)	-0.45(-0.97 to -0.06)	0.083		
6 mo	1.75(1.59)	2.21(1.83)	-0.62(-1.30 to -0.06)	0.075		
12 mo	1.73(1.60)	3.00(1.97)	-1.62(-2.31 to -0.92)	＜0.001		
**SF-36**						
Baseline	110.17(12.76)	113.42(12.01)	-3.36(-8.17 to 1.45)	0.168	3.376	0.019
3 mo	117.10(16.04)	117.08(14.32)	-0.51(-6.63 to 5.62)	0.870		
6 mo	118.81(15.77)	116.22(15.53)	2.65(-3.78 to 9.08)	0.415		
12 mo	121.51(14.21)	116.28(18.17)	-5.94（-0.77 to 12.64）	0.082		

© 2021 Peng MS et al. *JAMA Network Open.*

**SAS**						
Baseline	42.05(8.64)	42.23(9.79)	0.15(-3.45 to 3.75)	0.934	2.315	0.080
3 mo	37.23(8.69)	44.18(15.25)	-5.70(-10.61 to -0.80)	0.023		
6 mo	42.70(10.50)	43.54(12.01)	-1.06(-5.73to 3.60)	0.652		
12 mo	40.80(9.67)	40.54(11.07)	-0.74(-4.96 to 3.48)	0.728		
**SDS**						
Baseline	41.82(9.07)	43.63(9.84)	-1.91(-5.77 to 1.95)	0.329	0.784	0.503
3 mo	40.64(11.03)	44.49(12.97)	-3.88(-8.71 to 0.94)	0.113		
6 mo	41.91(11.74)	47.28(14.13)	-5.45(-10.81 to -1.00)	0.046		
12 mo	42.02(10.47)	45.35(14.36)	-3.82(-8.94 to -1.30)	0.142		
**PSQI**						
Baseline	7.04(3.45)	6.91(3.50)	0.28(-1.10 to 1.67)	0.686	3.490	0.019
3 mo	5.64(3.29)	6.89(4.15)	-1.25(-2.76 to 0.26)	0.103		
6 mo	6.66(3.92)	6.60(4.96)	0.22(-2.00 to 1.57)	0.811		
12 mo	6.13(2.76)	7.04(3.65)	-1.20(-2.50 to -0.11)	0.072		
**PASS**						
Baseline	27.77(12.25)	27.33(12.77)	0.78(-3.88 to 5.43)	0.742	3.590	0.016
3 mo	20.14(11.15)	26.05(16.33)	-5.30(-10.78 to -0.18)	0.058		
6 mo	19.36(12.23)	24.58(13.84)	-6.95(-11.98 to -1.93)	0.007		
12 mo	22.36(20.23)	22.75(15.02)	-0.97(-7.96 to 6.02)	0.784		
**TSK**						
Baseline	44.82(5.70)	42.30(4.99)	2.87(0.84 to 4.90)	0.006	5.082	0.003
3 mo	40.14(7.06)	41.51(6.04)	-1.23(-3.82 to 1.37)	0.350		
6 mo	38.93(9.17)	41.37(6.21)	-3.16(-6.23 to 0.09)	0.044		
12 mo	39.27(8.57)	41.23(6.02)	-2.34(-5.24 to -0.56)	0.113		
**FABQ**						
**FABQ-PA**						
Baseline	12.29(4.34)	12.58(4.14)	-0.37(-2.06 to 1.33)	0.669	2.309	0.076
3 mo	9.63(5.26)	11.84(5.10)	-2.31(-4.37 to -0.25)	0.028		
6 mo	9.50(5.03)	11.25(5.75)	-2.39(-4.55 to -0.23)	0.030		
12 mo	8.09(5.07)	10.96(6.05)	-3.18(-5.43 to -0.93)	0.006		
**FABQ-W**						
Baseline	25.70(9.46)	24.32(9.16)	2.00(-1.64 to 5.64)	0.279	1.774	0.152

© 2021 Peng MS et al. *JAMA Network Open.*

3 mo	21.96(11.94)	23.60(12.64)	-1.41(-6.25 to 3.44)	0.567		
6 mo	22.05(9.75)	24.93(13.96)	-3.21(-8.09 to 1.68)	0.196		
12 mo	20.66(11.01)	22.86(11.81)	-1.46(-6.00 to 3.08)	0.525		
**FABQ-TOTAL**						
Baseline	37.98(11.83)	36.89(11.48)	1.63(-2.90 to 6.16)	0.476	2.184	0.090
3 mo	31.59(16.25)	35.44(16.22)	-3.72(-10.10 to 2.66)	0.250		
6 mo	31.55(13.55)	36.18(18.74)	-5.60(-12.20 to 1.00)	0.096		
12 mo	28.75(15.21)	33.82(16.03)	-4.64(-10.86 to 1.58)	0.142		

Abbreviation: Roland Morris disability questionnaire (RMDQ), Numeric Rating Scale(NRS), Short Form Health Survey(SF-36), Self-Rating Anxiety Scale (SAS), Zung Self-Rating Depression Scale (SDS), Pittsburgh Sleep Quality Index (PSQI), Pain Anxiety Symptoms Scale (PASS), Tampa Scale for Kinesiophobia (TSK), Fear Avoidance Beliefs Questionnaire（FABQ）

[a] Mean difference between groups were adjusted for gender, age, BMI, physical activity, LBP duration, NRS of the most severe LBP, medication, and smoking history

© 2021 Peng MS et al. *JAMA Network Open.*
