# Supplementary material for: Efficacy of Therapeutic Aquatic Exercise vs Physical Therapy Modalities for Patients With Chronic Low Back Pain: A Randomized Clinical Trial
Source: JAMA Netw Open. 2022 Jan 7;5(1):e2142069. doi: 10.1001/jamanetworkopen.2021.42069 (PMC8742191; doi:10.1001/jamanetworkopen.2021.42069)
Supplement: Supplement 3. — Data Sharing Statement [file jamanetwopen-e2142069-s003.pdf]

Data Sharing Statement

Peng. Efficacy of Therapeutic Aquatic Exercise vs Physical Therapy Modalities for Patients With Chronic Low Back Pain. *JAMA Netw Open*. Published January 07, 2022. doi:10.1001/jamanetworkopen.2021.42069

Data
Data available: No
